# Redox Regulation of Complement Pathway Activation in Aging and Related Diseases

**DOI:** 10.3390/antiox15010029

**Published:** 2025-12-24

**Authors:** Shirin Ferdowsi, Srividya Arjuna, Sudharshan SJ, Rahima Zennadi

**Affiliations:** Department of Physiology, The University of Tennessee Health Science Center, 71 S. Manassas St., Memphis, TN 38163, USAssetraja@uthsc.edu (S.S.)

**Keywords:** oxidative stress, aging, complement system, complement activation, oxidative damage, reactive oxygen species

## Abstract

Aging is a complex degenerative process characterized by the accumulation of molecular damage and a heightened susceptibility to disease. The oxidative stress theory of aging identifies endogenous reactive oxygen species (ROS) as primary drivers of this cellular deterioration. This review provides a comprehensive analysis of the critical, yet underappreciated, interplay between oxidative stress and the complement system, a powerful effector of innate immunity. We detail the mechanistic pathways through which redox imbalance directly activates complement components and, conversely, how complement activation amplifies oxidative stress, creating a vicious cycle that accelerates tissue damage. A central focus is placed on how this redox–complement axis contributes to the pathophysiology of age-related conditions, including neurodegenerative, cardiovascular, and metabolic diseases. Furthermore, the review explores emerging therapeutic strategies that target this interaction, highlighting the potential of antioxidant and complement-inhibitory approaches to disrupt this cycle and promote healthy aging. By synthesizing current evidence, this work underscores the significance of the redox–complement network as a key mechanistic link in aging and its associated diseases.

## 1. Introduction

Aging is an irreversible, multifactorial process driven by molecular alterations that progressively reduce cellular function, leading to organ decline and heightened vulnerability to disease [1]. While numerous theories attempt to explain aging, Denham Harman’s oxidative stress theory (1956) remains prominent, proposing that ROS generated as metabolic by-products inflict cumulative damage on applicable cellular constituents [2]. In health, a balance between oxidants and antioxidants prevents this damage, but this equilibrium is disrupted with age [3,4].

Parallel to oxidative stress, the complement system is increasingly recognized as a critical contributor to age-related pathology. As a cornerstone of innate immunity, complement defends against pathogens and clears cellular debris, but its dysregulation can provoke significant tissue injury [5]. Complement activation is a tightly regulated cascade involving classical, lectin, and alternative pathways, converging on the formation of effector molecules like the anaphylatoxins (C3a, C5a) and the membrane attack complex (MAC) [6,7,8].

Emerging evidence indicates that oxidative stress is a potent trigger of complement activation, particularly in vascular and ischemic injury models [9,10,11,12,13]. This review synthesizes evidence establishing oxidative stress as a key activator of the complement system in aging. We examine the mechanisms of this interaction, its role in disrupting homeostasis, and its contribution to the pathogenesis of various age-related diseases, proposing that this crosstalk represents a fundamental mechanism underlying the aging process.

In summary, this review will critically analyze the interconnected roles of oxidative stress and dysregulated complement activation as key drivers of the aging process and the pathogenesis of age-related diseases. It will explore the molecular mechanisms by which ROS potentiate complement pathway activity and, conversely, how complement activation contributes to a pro-oxidant environment, creating a vicious cycle that accelerates cellular senescence, tissue damage, and functional decline. Furthermore, the review will discuss the implications of this redox–complement axis for the development of novel therapeutic strategies aimed at interrupting this cycle to promote healthy aging and mitigate the burden of conditions such as neurodegenerative disorders, cardiovascular disease, and other senescence-associated pathologies.

## 2. Free Radical Theory of Aging and Sources of ROS

Free radicals are highly reactive molecules produced naturally at low concentrations, where they support essential cellular functions and defense mechanisms. A free radical is defined as an atom or molecule with one or more unpaired electrons, typically formed when oxygen interacts with specific molecules [14]. They arise primarily through redox reactions involving electron transfer (oxidation–reduction) or through homolytic cleavage of covalent bonds, processes that generate electron donors and acceptors collectively termed pro-oxidants [15,16]. The reaction chain begins with the single-electron reduction of oxygen to form superoxide (O_2_ + e^−^ → O_2_^−^), which rapidly dismutates to hydrogen peroxide (2O_2_^−^ + 2H^+^ → H_2_O_2_ + O_2_). Hydrogen peroxide can then be converted into the highly destructive hydroxyl radical via the Fenton reaction (H_2_O_2_ + Fe^2+^ → OH^·^ + OH^−^ + Fe^3+^) or form peroxynitrite with nitric oxide (NO^·^ + O_2_^−^ → ONOO^−^).

As per the free radical theory of aging, increasing cellular damage from free radicals exacerbates the aging process [17]. Although antioxidant defenses such as catalases, superoxide dismutase (SOD), and glutathione peroxidases (GPXs) normally mitigate oxidative stress, they become less efficient with advancing age, leading to progressive accumulation of oxidatively damaged molecules. ROS include hydroxyl radicals (OH^·^), superoxide anions (O_2_^·^^−^), and hydrogen peroxide (H_2_O_2_), which differ in stability, with O_2_^·^^−^ and OH^·^ being highly unstable and H_2_O_2_ comparatively long-lived [18]. Mitochondria are a major source of ROS [19,20,21], but cytosolic enzyme systems also contribute significantly, including NADPH oxidases, cytochrome P450, xanthine oxidase (XO), and uncoupled nitric oxide synthase (NOS) [22,23]. Among these, NADPH oxidases predominate in vascular cells, where they drive vascular dysfunction and age-related pathologies [24,25,26]. Their activation is stimulated by diverse factors such as hypoxia, mechanical stress, cytokines, and hormones (e.g., angiotensin II, platelet-derived growth factor, endothelin-1, TGFβ, TNFα) [27,28,29,30,31,32,33,34,35]. Our recent data support this, showing that ROS production in red blood cells (RBCs) from mid-life adults is NADPH-oxidase-dependent [27], highlighting these enzymes as central to oxidative stress in aging and potential therapeutic targets.

Importantly, dysregulated complement activation intersects with these pathways and acts as a critical amplifier of oxidative stress during aging. The complement system, a central arm of innate immunity, when chronically overactivated, fosters both oxidative stress and persistent inflammation, two hallmarks of aging-related diseases. Complement-mediated mechanisms include mitochondrial dysfunction, where ROS leakage impairs energy metabolism and accelerates cellular injury; blood–brain barrier breakdown, which increases susceptibility to neurodegenerative disorders such as Alzheimer’s disease; and cellular senescence, where senescent cells release pro-inflammatory cytokines (IL-6, TNF-α) and ROS that exacerbate tissue damage. Sustained complement activity also promotes “inflammaging,” a state of chronic low-grade inflammation, through continuous immune cell recruitment and ROS overproduction by neutrophils and macrophages. Together, these processes establish a vicious cycle in which complement-driven inflammation and excessive ROS reinforce each other, accelerating tissue injury and age-related disease progression.

## 3. Activation of the Complement System

**Figure 1:**
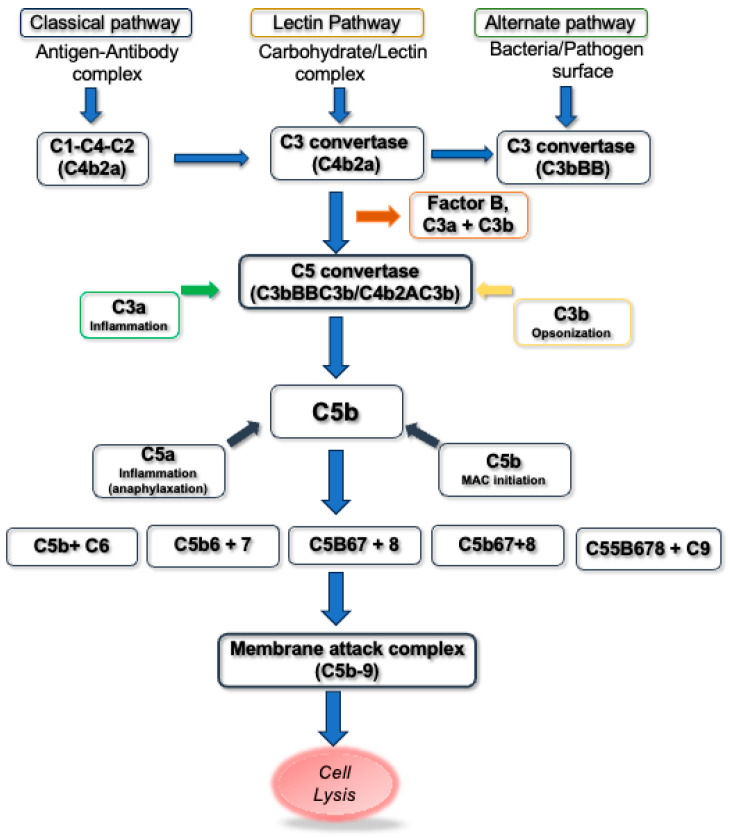
Pathways of complement activation showing the formation and cleavage of C3 and C5 complement components with C3 and C5 convertase, respectively, leading to the formation of C3a and C5a anaphylatoxins and formation of MAC.

The complement system is recognized as a powerful part of the cytotoxic defense system to eliminate foreign cells and initiate inflammation. The complement system recognizes not only the molecular patterns on foreign pathogens but also cell debris and unwanted host cells to be removed by phagocytosis [36]. The complement system comprises three main pathways—the classical, the lectin, and the alternative pathways (CP, LP, and AP, respectively) (Figure 1). The activation of these pathways occurs through a cascade of proteolytic cleavage. The activation of the classical pathway depends on antibodies. The antigen–antibody complexes interact with the complex protein C1. C1 is a complex composed of a large subunit C1q and two esterase subcomponents C1r and C1s. Binding of C1q to the Fc region of complexed immunoglobulins results in activation of C1r and C1s esterases, followed by cleavage of C4 and C2 complement components leading to the formation of C3 convertase [37]. The lectin pathway of the complement cascade is an antibody-independent process initiated by the binding of mannose-binding lectin (MBL) to the carbohydrates on the cell surface [38]. MBL shows structural similarity to that of C1q and may anticipate C1q evolutionarily [39]. It is associated with two serine proteases known as MBL-associated serine proteases, MASP-1 and MASP-2 [40]. Upon activation, these two serine proteases cleave C2 and C4 complement components, resulting in classical pathway C3 convertase [41]. Unlike the classical and lectin pathways, components of the alternative pathway are unique; they include factor B, factor D, and properdin. They are capable of autoactivation as a result of “tickover” of C3. “Tickover” results in conformationally different C3, known as C3(H_2_O), capable of binding to factor B. Association of factor B with C3(H_2_O) results in conformational changes and it is cleaved by factor D serum protease, leading to the generation of Bb and Ba. The Bb fragment thus formed remains linked to the complex and cleaves additional C3 molecules by its own serine protease. The C3 complement components form a C3b fragment and this gets associated with factor B to form more C3 convertase. The entire cascade process is enhanced by the serum protein properdin that stabilizes protein–protein interactions [42].

Activation of the three complement pathways converges eventually at the level of generation of C3 and C5; thereafter the three pathways continue as a common reaction process through the late complement components C6–C9, resulting in the formation of the membrane attack complex (MAC), which is the main effector of complement-mediated tissue damage [6,7,8]. The MAC is a transmembrane channel which allows the influx of water and salt, resulting in osmotic lysis of MAC-targeted cells. The formation of the MAC channel represents the end phase of the complement process, resulting in significant changes in cell membrane permeability and viability of the MAC-targeted cells [43,44,45].

Complement C5 activation is typically mediated by enzymatic convertase complexes; however, elevated reactive oxygen species (ROS) can also induce non-enzymatic cleavage of C5. This alternative cleavage generates C5a-like (C5a·) and C5b-like (C5b·) fragments, which in turn promote membrane attack complex (MAC) assembly, inflammation, and cell lysis.

$$C5+\mathrm{ROS}\overset{\mathrm{Oxidative damage}}{\to }C5a+C5b\to \mathrm{MAC}\;\mathrm{formation}\to \mathrm{Inflammation}\to \mathrm{Cell}\;\mathrm{lysis}$$

This intricate activation cascade, while essential for host defense, becomes a potent source of pathological inflammation and tissue damage when dysregulated—a vulnerability that is critically exacerbated in the context of aging and oxidative stress, as explored in the following section.

## 4. Regulators of Complement Activation

**Figure 2:**
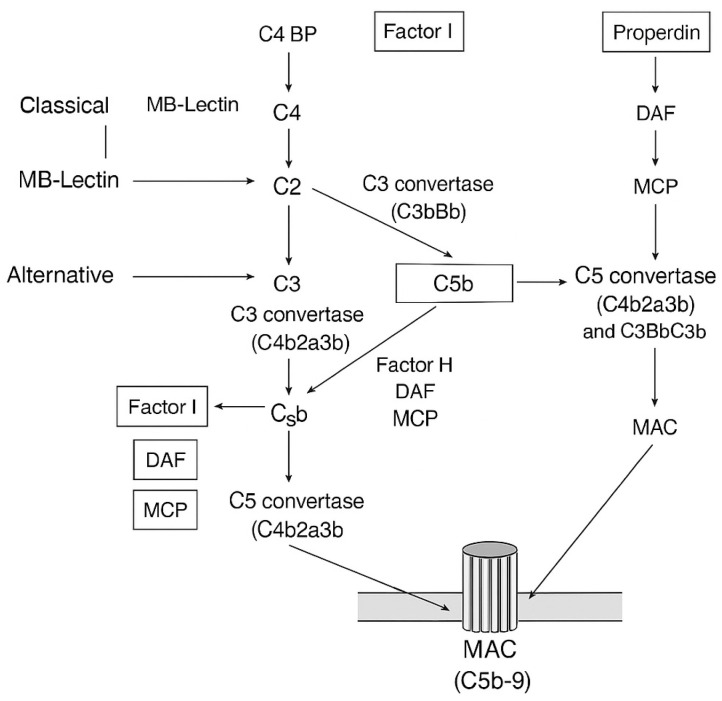
This schematic diagram illustrates the mechanisms through which complement regulators control the activation, amplification, and effector functions of the complement system. It depicts both inhibitory and activating components to demonstrate how regulatory proteins help maintain a balance between protecting the host and preventing excessive inflammatory responses.

As the uncontrolled complement activation has a potential devastating effect on body homeostasis, an array of defense mechanisms has progressed to rescue “self” cells from MAC formation and complement attack [45]. To safeguard host tissues, the complement system is tightly controlled by multiple regulatory proteins and cellular defenses. Soluble regulators in plasma such as factor H (FH), factor I, and C1 inhibitor (C1-INH) dampen complement activity at the initiation and amplification stages, while vitronectin and clusterin specifically block MAC insertion (Figure 2). At the cellular level, membrane-bound regulators including CD46, CD55, CD59, and complement receptor type 1 (CR1) act directly on host cell surfaces to prevent complement-mediated injury by promoting convertase decay, inactivating opsonins, or blocking MAC assembly. In addition, cellular and metabolic mechanisms—such as expression of complement inhibitors by endothelial and immune cells, shedding of complement-binding sites, and anti-inflammatory cytokine production—further restrain excessive activation. Together, this multilayered defense network ensures effective pathogen clearance while preserving self-tolerance and tissue integrity.

Despite these protective measures, healthy host cells can still become inadvertently tagged by C3b—either through spontaneous hydrolysis of C3 [C3(H_2_O)] that triggers alternative pathway activation or via a “bystander” effect where C3b generated nearby attaches to host surfaces if not properly regulated [46,47]. This underscores the need for precise discrimination between self and non-self, a task carried out by key complement regulators. Among the most studied, FH [48] distinguishes host from pathogen by recognizing host-specific glycans, thereby limiting complement activation on self-surfaces through decay-accelerating activity that disrupts alternative pathway C3 convertase [49]. Similarly, C1-INH restrains the classical and lectin pathways by inactivating C1r, C1s, MASP-1, and MASP-2, while also regulating contact system enzymes such as kallikrein and factor XIIa (FXIIa) [50]. On cell membranes, CD59 prevents MAC formation by blocking C9 incorporation into C5b-8 complexes [51] and eliminating nascent MACs [52], while CD46, CR1, and CD55 further protect host cells by inactivating opsonins, accelerating convertase decay [52,53,54,55], and contributing to immune complex clearance.

## 5. Complement Activation, Oxidative Stress, and Aging

Disproportionate activation of the complement system results in organ injury and contributes to a wide range of pathological conditions. These disorders can be categorized by their underlying mechanisms: genetic defects in complement regulation (e.g., atypical hemolytic uremic syndrome (aHUS), paroxysmal nocturnal hemoglobinuria (PNH)), immune-complex-driven diseases (systemic lupus erythematosus (SLE), rheumatoid arthritis (RA)), dysregulation-associated conditions (age-related macular degeneration (AMD)), acute inflammatory states (sepsis, ischemia–reperfusion injury), and transplantation-related pathology (graft rejection). Together, these categories highlight how complement, though essential for host defense, becomes a pathogenic driver when its regulation fails.

Beyond genetic or immune triggers, the complement system plays a central role in mediating tissue injury under oxidative stress conditions. ROS can directly activate complement components and promote their deposition on vascular endothelium [9,10,11,12], thereby disrupting vascular homeostasis. In ischemia–reperfusion injury, oxidative stress amplifies complement activation and alters endothelial balance [56,57], fueling inflammation and tissue damage. Experimental studies demonstrate that complement inhibition [53,58,59,60,61], depletion [53,62,63], or genetic deficiency [64,65,66] significantly reduces ischemia/reperfusion-mediated injury, preserves endothelium-dependent relaxation [62,63], and diminishes pro-inflammatory cytokine secretion [66], underscoring the contribution of complement to oxidative vascular injury. Historically, Hill and Ward showed that ischemic myocardium produces proteases capable of cleaving C3, leading to leukocyte activation and chemotaxis [67]. Subsequent work confirmed deposition of C1q, C3, C4, C5, and C5b-9 complexes in human and animal models of myocardial ischemia [66,68]. Importantly, oxidative stress serves as both a trigger and amplifier of this response: hydrogen peroxide and other ROS have been shown to activate C5 via non-enzymatic mechanisms [37], while inhibition of ROS formation reduces complement activation and deposition [27,69].

Mechanistically, complement activation under oxidative stress involves both antibody-dependent and antibody-independent pathways. The classical pathway may be engaged through antibodies binding to neo-epitope [70] formed during oxidative injury, as immunoglobulin-deficient mice are protected against hindlimb ischemia-mediated vascular injury, with protection lost upon antibody reconstitution [70]. However, endothelial cells under anoxic conditions also show complement activation in the absence of immunoglobulins, highlighting antibody-independent mechanisms [71]. The lectin pathway is particularly relevant, as oxidative stress and hypoxia can alter cell-surface glycosylation, enhancing MBL recognition of endothelial proteins [57,72,73]. Notably, oxidative stress increases expression of cytokeratin-1, which promotes MBL binding and subsequent C3 deposition [74]. Inhibition of MBL reduces complement activation triggered by cytokeratin and oxidative stress [74], suggesting that MBL–cytokeratin interactions are a key mechanism linking ROS and lectin pathway activation.

In the context of aging, these mechanisms acquire heightened significance. Dysregulated complement activation contributes to mitochondrial dysfunction [75,76], cellular senescence [77], blood–brain barrier breakdown [78], and chronic low-grade inflammation (“inflammaging”) [76,78]. Complement activation products, particularly C3a and C5a, recruit neutrophils and macrophages, leading to sustained ROS production [78,79]. This establishes a vicious cycle in which oxidative stress amplifies complement activation and vice versa [76,80], resulting in progressive damage to lipids, proteins, and DNA in aging tissues. Thus, complement is not only a mediator of acute ischemic injury but also a central amplifier of oxidative stress and chronic inflammation that underpins age-related neurodegenerative, cardiovascular, and metabolic diseases [76,78,80].

## 6. The Complement System and Homeostasis

Normally, in the bloodstream, the complement system forms close contact with the platelets, coagulation factors, and fibrinolytic system contributing to various biological functions. These systems function together as a first line of defense against foreign pathogens entering the blood stream and initiate tissue repair upon damage, either causing sustainable homeostasis or resulting in severe complications [81].

### 6.1. The Role of Platelets in Regulating the Complement System

**Figure 3:**
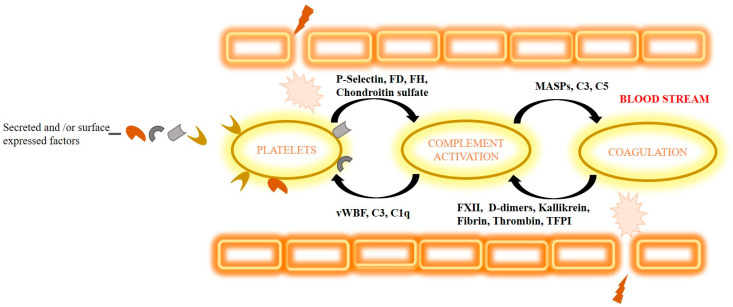
Association of platelets and coagulation system with the complement. Upon injury, several platelet and coagulation factors activate resulting in activation of complement components leading to initiation of complement pathways.

Platelets play a key role in regulating complement activation through several secreted and/or surface-expressed factors (Figure 3). For initiation of complement activation, platelet-expressed P-selectin triggers the complement cascade either on its own or by spontaneous basal plasmatic cleavage of C3 and by fixing C3b [82,83,84]. By producing chondroitin sulfate, platelets bind C1q or factor D (FD) resulting in the initiation of local complement activation [85,86,87]. Regulation of complement activation by platelets is carried out by secreting factor H (FH) and regulating the activity of C3 convertase or by modulating the effect of C1q through CR3 [88,89]. Factor H also mediates regulation of the complement classical pathway activation by fibrin clots [90]. Another new regulator mechanism of complement activation is von Willebrand factor (vWF)-multimers causing platelet adhesion. vWF is suggested to down-regulate complement activation and guard endothelial cells from complement-mediated injury [91]. By secreting chondroitin sulfate, platelets bind complement regulators factor H, C1 inhibitor (C1INH), and C4b-binding protein (C4BP), thus hindering activation of the complement cascade on its surface [81,92].

### 6.2. The Role of the Coagulation Cascade in Regulating the Complement System

The coagulation cascade and the complement system are associated in terms of activation mechanisms and influence functions of innate immunity upon tissue injury [93]. The initiation of complement activation by coagulation factors is carried out in several ways (Figure 3). Factor XII (FXII) binds to complement component C1 or globular C1q receptor (gC1qR) resulting in the initiation of activation of the alternative pathway [94,95]; plasma fibrin and plasmin-generated fibrin fragments (D-dimers) bind and stimulate MASP-1 and MASP-2 complement components, leading to activation of the lectin pathway; and cleavage of factor B and activation of C1s by pre-kallikrein result in activation of both the classical and the alternative pathways [96]. In the meantime, kallikrein not only cleaves factor B but C3 and C5 complement components as well [97,98,99]. The association of fibrin/fibrinogen with MASPs leads to the activation of C3 and C4 complement components and surface deposition of C3b and C4b [100]. An in vivo study has shown the cleavage of C5 in the absence of C3 by thrombin [101]. Further study has discovered that cleavage of C3 and C5 complement components, resulting in C3a and C5a, respectively, was possible by several other factors like FIXa, FXa, FXIa, and plasmin besides thrombin. Moreover, the tissue factor pathway inhibitor and thrombomodulin contribute to regulation of the complement system [72,102,103,104]. Apart from these mechanisms, NETosis of neutrophil extracellular traps (NETs) seems to be another important mediator of complement–coagulation interaction in protecting the host against foreign pathogens. During coagulation, NETs act as a platform for thrombus formation [105].

### 6.3. Crosstalk Between Mitochondria-Specific ROS Production in Aging and Activation of the Complement System

Among several cell organelles that could potentially engage through functional crosstalk with complement or be influenced by complement receptors, mitochondria are amongst the main contenders [106]. Proper integrity of mitochondrial DNA (mtDNA) is essential for proper functioning of mitochondria. Age-related oxidative-stress-induced variations in mitochondrial gene expressions may result in abnormal functioning of mitochondrial subunits leading to diseases [107]. Mutations in mtDNA may lead to a vicious event wherein abnormal functioning of mitochondria contributes to the formation of ROS, resulting in increased ROS-mediated oxidative damage to the mitochondria [106,108,109,110,111]. Moreover, variations in the synthesis of coenzyme Q may modify mitochondria-specific ROS production particularly through reverse electron transport; thus correlating with mitochondrial diseases [112]. Several studies have suggested that mitochondrial dysfunction is precisely related to the aging phenotype and evolves more rapidly [113,114,115,116,117] and is associated with pathogenesis of age-associated diseases like Alzheimer’s disease [118]. Mitochondrial mutations that impair ATP production further accelerate premature aging and neurodegeneration [117,119,120].

The theory of mitochondria-specific ROS production in aging has been refined and debated over the decades, but two principles remain broadly accepted: (i) oxidatively damaged molecules accumulate with age due to imbalance between pro-oxidant and antioxidant agents, and (ii) aging-associated degeneration is driven by this cumulative oxidative damage [121]. Additional mitochondrial enzymes, including the α-ketoglutarate dehydrogenase complex and monoamine oxidase, significantly contribute to ROS production in a tissue-specific manner [122,123,124,125]. Thus, mitochondrial activity and ROS generation are inseparably linked, with mitochondria accounting for ~90% of total intracellular ROS [108,121,126].

A further layer of complexity arises from crosstalk between mitochondria and the complement system. Early studies reported that the mitochondrial membrane harbors binding sites for complement C1q, suggesting that C1q–mitochondria interactions can initiate complement C1 activation [127]. Binding and activation of C1 by mitochondrial or subcellular membranes may enhance inflammatory processes following acute tissue injury, and mitochondrial localization has been reported for complement components C1q, C3, C5, and C9 [128]. For instance, C1q can accelerate mitochondrial ROS emission, exacerbating oxidative injury in the hypoxic–ischemic brain [129]. Certain phospholipids also facilitate antibody-independent C1 activation by mitochondria, further amplifying complement responses [127]. Although our understanding of this relationship is still emerging, the evidence points to complement as a key nexus linking redox imbalance, mitochondrial dysfunction, and aging.

## 7. The Role of Oxidative-Stress-Induced Complement Activation in Triggering the Onset of the Aging Process

Studies have suggested that the genetic inheritance of aging appears to be only 3%, while epigenetic and post-translational alterations are the key factors contributing to aging [130,131,132]. Aging could also be linked to mitochondrial dysfunction. For instance, reduced oxidative phosphorylation leads to increased production of ROS. This in turn activates p66Shc, a pro-apoptotic protein involved in ROS production in mitochondria, subsequently inducing further ROS production, which results in apoptosis and sustaining a stable aging process. Hence, p66Shc protein may serve as a key factor connecting ROS and aging [133]. Furthermore, oxidative-stress-induced damage has been found to be one of the contributing factors for telomere shortening and damage leading to the aging process and development of age-associated diseases [130,134,135,136,137]. The genetic relationship between stress responsiveness and longevity has been well studied in *C. elegans* and mice [138,139]. The significant overlap between genes governing stress resistance and those influencing lifespan demonstrates that longevity is intrinsically linked to an organism’s ability to withstand stress. This overlap also underscores the role of the innate immune system as a key regulator of lifespan, with common genetic factors shaping both immune defense and longevity. Specific gene mutations like *mev-1* and *ctl-1*, resulting in reduced lifespan, seemed to be associated with oxidative stress, and this notion is well described in *C. elegans*. The gene *mev-1* codes for a subunit of enzyme succinate dehydrogenase cytochrome b of the electron transport chain in mitochondria, while *ctl-1* codes for catalase in the cytosol [140]. In *C. elegans*, mutations of the electron transport chain also modulate lifespan and ROS production [141]. In addition, the rapid aging observed in Sod1^−/−^ mice is associated with elevated levels of cellular senescence [142]. Further support for the association of stress tolerance and longevity in *Drosophila* has been found by overexpressing antioxidant genes using transgenic approaches. Increased glutathione and thioredoxin reductase extend the life expectancy of flies against dichlorvos [143]. Some studies have shown increased expression of CuZn-SOD or in association with catalase, likely showing enhanced stress resistance and increased life expectancy [144,145,146,147]. Yet, some other studies conveyed little or no enhanced life expectancy or enhanced stress resistance with increased expression of CuZn-SOD [148]. Thus, the source for these inconsistencies still remains unclear. The consequence of increased SOD expression on stress responsiveness and longevity has also been studied in mice. However, many studies reported increased CuZn-SOD levels as a defense mechanism against acute oxidative stress. One of the studies by Huang et al. indicated that this defense mechanism did not show correlation with increased longevity [149].

The relationship between aging and ROS production is non-linear, with different levels of oxidative stress exerting opposing effects. At low levels, ROS can protect cellular structures by activating defense systems, whereas high levels promote oxidative damage and accelerate the aging phenotype [150]. The precise mechanisms underlying cellular aging remain unclear and warrant further investigation. Beyond oxidative stress, DNA damage, mitochondrial dysfunction, oncogene activation, and the loss of tumor suppressor gene function also contribute to cellular aging [151]. Consequently, the original oxidative stress theory of aging was reconsidered in 2014 [152], as multiple studies demonstrated that the impact of ROS on aging depends on genotype, metabolic state, and the efficiency of antioxidant defenses [145,153,154,155].

**Figure 4:**
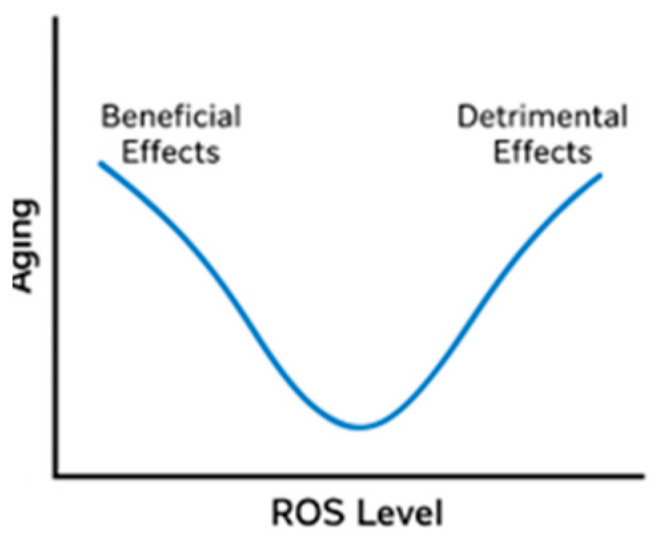
The non-linear relationship between aging and ROS.

This non-linear nature arises because ROS can be both beneficial and detrimental (Figure 4), depending on concentration. At low to moderate levels, ROS act as signaling molecules regulating proliferation, differentiation, and stress responses. They activate protective pathways such as NRF2, inducing antioxidant defenses and promoting longevity. This adaptive response, termed mitohormesis, suggests that modest ROS exposure is essential for homeostasis and lifespan extension. By contrast, excessive ROS cause oxidative stress, leading to irreversible damage to DNA, proteins, and lipids, thereby accelerating senescence and age-related disease. Thus, the ROS–aging relationship follows a U- or inverted J-shaped curve: both insufficient and excessive ROS are harmful, whereas optimal levels support resilience and healthy aging.

Caloric restriction, which extends lifespan in multiple species including rodents, provides further evidence for this model. It reduces the incidence of age-associated diseases [156], prevents age-related alterations in gene expression, and modulates transcriptional activity. For example, caloric restriction maintains basal expression of heat-shock proteins [157,158] while reducing stress-induced Hsp70 expression [159], both of which typically change with aging. Environmental factors can also accelerate aging, a process known as exogenous aging [160]. Chronic infections, UV radiation, heavy metal exposure, and smoking all enhance oxidative stress and promote age-related decline [160].

**Figure 5:**
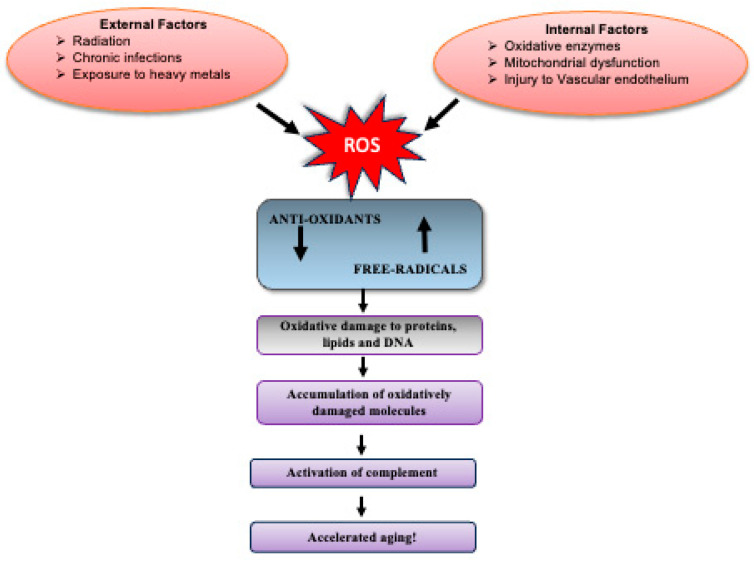
Factors triggering the aging process: Sources of ROS generation leading to oxidative-stress-mediated complement activation and accelerated aging. ↓ indicates decrease and ↑ indicates increase.

Oxidative stress also links aging with complement activation. Complement component C1q increases with age and activates canonical Wnt signaling, driving age-related declines in tissue regeneration in mice [161]. Complement C3a impairs proteasome activity in human retinal pigment epithelial cells and in animal models of age-related degeneration, thereby compromising protein quality control [162]. Moreover, activation of C3a and C5a receptors (C3aR and C5aR) has been implicated in regulating telomerase activity, preserving telomere length in cardiac resident stem cells [163]. Together, these findings suggest that oxidative stress, whether from endogenous or exogenous sources, accelerates aging in part through complement activation. As illustrated in Figure 5, nutritional, pharmacological, and environmental strategies that reduce ROS and modulate complement activation may therefore slow the aging process [164].

We are able now to link oxidative stress to complement activation during aging [27]. We found that RBCs of mid-life stage adults from 55–65 years old have higher ROS production than RBCs of a younger cohort (21–30 years old). Increased RBC ROS generation involved NADPH oxidases, Nox1 in particular, and G protein-coupled receptor kinase 2 (GRK2). This deleterious mechanism activated in RBCs of the mid-life stage population mediates complement system (C3 and the activated form anaphylatoxin C3a) deposition on RBCs. The inflammatory cytokine TGF-β1 operates directly via the RBC GRK2/NADPH oxidase/ROS pathway to exacerbate the complement C3 and C3a deposition on the RBC surface. ROS-dependent C3/C3a deposition on RBCs of mid-life stage adults triggered not only activation of prothrombin but also increased endothelial oxidative stress and damage. Using human RBCs and *Rpl13a* snoRNA knockout (KO) aged mice, we show that *Rpl13a* snoRNAs regulate RBC C3a deposition and prothrombic activation in aging [27]. *Rpl13a* snoRNA KO in aged mice reduced RBC Nox1 mRNA and protein expression and thrombus size by blunting C3a deposition and RBC-elicited prothrombin activation. These findings point to not only a broader role for the ROS in mediating complement C3/C3a deposition on RBCs and activation of the alternative pathway in aging but also to a novel role of RBC *Rpl13a* snoRNAs in dysregulating RBC ROS-induced C3a deposition promoting venous thrombosis in aging [27].

## 8. Oxidative Stress in Aging and Related Diseases

The hallmark of aging is a progressive decline in tissue and organ function. This is mainly due to the imbalance in the formation of ROS and antioxidant defense system altering the normal functioning of several tissues [165]. Moreover, several chronic diseases consequent of older age like diabetes, skeletal muscle disorders, and pulmonary, renal, and cardiovascular diseases are correlated with oxidative stress. The key feature of aging is accumulation of cellular senescence [166] encouraged by the damaging stimuli from inside and outside the cell [167]. Cellular senescence hampers the body tissue in two ways: one, the increasing accumulation of senescent cells hinders tissue regeneration; and two, secretion of increasing inflammatory factors and senescence-associated secretory phenotype (SASP) has a negative consequence on the adjacent environment [166,167]. Therefore, maintaining good cellular balance is of utmost importance. As per the reports from Global Burden of Diseases in 2017, 31.4% of diseases (92 of 293) were found to be age-related. Some of the most common age-related disorders include cardiovascular diseases, neurodegenerative diseases, cancer, osteoarthritis, osteoporosis, hypertension, and various chronic conditions.

### 8.1. Oxidative Stress and Cardiovascular Diseases

Cardiovascular disease remains the leading cause of deaths in the elderly, with atherosclerosis being the main causal event [168]. Studies have shown that, with advancing age, the tolerance of the heart to oxidative stress gradually decreases with the reduction in antioxidant enzyme concentrations, contributing to cardiovascular alterations [169]. Generally, cardiovascular tissues establish various pathological variations with advancing age; some of them include altered left ventricular diastolic function, decreased left ventricular systolic standby capacity, hypertrophy, enhanced arterial stiffness, and abnormal endothelial function [170]. However, increased oxidative stress leads to vascular endothelial dysfunction with aging. Moreover, atherosclerosis leads to inflammation and further changes in the vasculature thereby increasing risk for cerebrovascular events, cardiac events, peripheral vascular disease, and other organ damage [171]. Vascular remodeling and endothelial dysfunction are considered as the two key elements in pathogenesis of hypertension and atherosclerosis [172].

### 8.2. Oxidative Stress and Neurodegenerative Diseases

Aging is one of the common factors leading to progressive neurodegenerative diseases. Alzheimer’s disease (AD) is considered as the most frequent form of neurodegenerative disease associated with increasing prevalence with advancing age [173]. Other important neurodegenerative diseases like Parkinson’s disease, Huntington’s disease, and other vascular dementias have a great impact on elderly populations. Oxidative stress is an important key factor responsible for the pathophysiology of dementia [174]. Several studies have assessed the relationship between oxidative stress biomarkers and neurodegenerative disease pathophysiology. One of these studies reported elevated oxidative stress biomarkers along with increased inflammatory cytokine level associated with decreased cognitive presentation in an institutionalized older population [175]. Likewise, brain aging is largely manifested by several pro-inflammatory factors distinguished by altered signaling, SASP, and accretion of senescent glia [176]. Many theories suggest the role of oxidative stress associated with the pathological process of neurodegenerative diseases. The role of ROS and redox metals has been suggested in the pathological process of Alzheimer’s disease. This is mainly because of altered levels of bioactive metals that may lead to oxidative stress thereby influencing Alzheimer’s disease pathogenesis. Metals like iron bind to amyloid, enhancing its accumulation and aggregation [177]. Another theory suggests that oxidative stress may induce formation of stress granules. Under pathological conditions, these stress granules may form super-stable masses that have a negative impact on their clearance [178].

### 8.3. Oxidative Stress and Cancer

Cancer is considered as the second main cause of death in older populations. However, it has been reported that the death rate from cancer subsides by 85 years [179]. Studies show a relationship between carcinogenesis, chronic inflammation, and free radical species as the chief chemical effectors of the inflammatory response [180]. Additionally, reactive oxygen and nitrogen species may harm other components in target cells or may pave the way to additional inflammatory cells, resulting in exacerbation in reactive oxygen and nitrogen production and thereby worsening the damage [181]. Some studies reveal that accretion of senescent cells leads to increased expression of SASP and promotes an inflammatory state resulting in enhanced invasive capabilities and accelerated cancer progression [182]. Chronic inflammation is linked with angiogenesis, as reactive oxygen and nitrogen species may contribute to an increase in the expression of transcriptional factors like c-jun and c-fos involved in enrichment of cancer angiogenesis and neoplastic transformation [181]. Epigenetic variations are key factors connecting the aging process and cancer [183]. Specifically, ROS-induced DNA damage may result in genomic instability, induction or replication errors, and transcriptional arrest which are more closely connected to cancer [184]. Amongst oxidized DNA products, 8-nitroguanine and 7,8-dihydro-8-oxo-2′-deoxyguanosine (8-oxo-dG) are given due consideration as biomarkers for inflammation-influenced carcinogenesis [180]. Interestingly, it is observed that, with advancing age, the DNA damage induced by reactive oxygen and nitrogen species increases which is validated by a substantial increase in the levels of 8-oxo-dG detected in aging [180]. Therefore, based on these considerations, oxidative stress and chronic inflammation may be measured as critical risk factors for cancer in the elderly population.

### 8.4. Oxidative Stress and Musculo-Skeletal Diseases

Aging is commonly associated with muscle strength discrepancies that are interrelated with physical disabilities and frailty [185]. Osteoarthritis and sarcopenia are considered as the most common age-related musculo-skeletal diseases having a substantial economic impact [186]. It is postulated that the skeletal muscles consume a great amount of oxygen, thereby producing a large quantity of reactive oxygen and nitrogen species. The accumulation of these free radicals is thought to be the main basis for loss of muscle quality and quantity [187]. Augmented inflammation may also contribute to further ROS production in skeletal muscles, leading to increased cell apoptosis and affecting catabolism of skeletal muscle [188]. With advancing age, the accumulation of free radicals increases and antioxidant defenses are compromised, which induces post-transcriptional variations like carbonylation (i.e., PC, 4-HNE), nitration and nitrosylation (i.e., NT), and glycation (i.e., AGEs) which can be used as oxidative stress biomarkers [189]. The observations made by spectral tracing experiments in mouse models have revealed that, due to aging, diminished proliferation activities of chondrocytes could be responsible for osteoarthritis progression [190]. Moreover, depleted antioxidant defenses and altered mitochondrial functions have been reported to be linked with progress of sarcopenia [191]. Reactive oxygen and nitrogen species also contribute to the pathological process of sarcopenia by enhancing proteolysis and diminishing protein synthesis in muscles, leading to lessening of muscle mass quantity [192].

### 8.5. Oxidative Stress and Multiform Chronic Conditions

About sixty two percent of Americans above the age of 65 years have more than one chronic condition [193]. Unfortunately, the prevalence is cumulative [194] due to advancing age in the population and increases in the rate of incidence of diabetes. Hypertension is the key contributor to the incidence of atherosclerosis, one of the more frequent chronic diseases in older populations [195]. On the other hand, diabetes is considered as the key risk factor for cardiovascular diseases in older populations [196]. It is also related to peripheral arterial disease and peripheral neuropathy, leading to severe disease and resulting in diabetic foot ulcers and amputations. Obesity, however, is the key risk factor causing osteoarthritis [197], one of the most frequent chronic conditions, with increasing severity with advancing age. Aging is also associated with dramatic changes in the immune system, resulting in diminished or loss of immunity to fight against foreign pathogens, infections, and cancer, thereby increasing the menace of autoimmune disorders [198]. The accumulation of CD28^−^ CD8^+^ T cells, which are found to be absent in neonates, constitutes about 80–90% circulating CD8^+^ T cells in elderly populations, thus representing one of the hallmarks of age-associated changes in the immune system [198]. Interestingly, the circulation of CD28^−^ CD8^+^ T cells has been observed and reported during viral infections like COVID-19, and this could explain the severity of COVID-19 observed in elderly populations [199].

## 9. Age-Associated Variations in Complement System Function and Related Diseases

**Figure 6:**
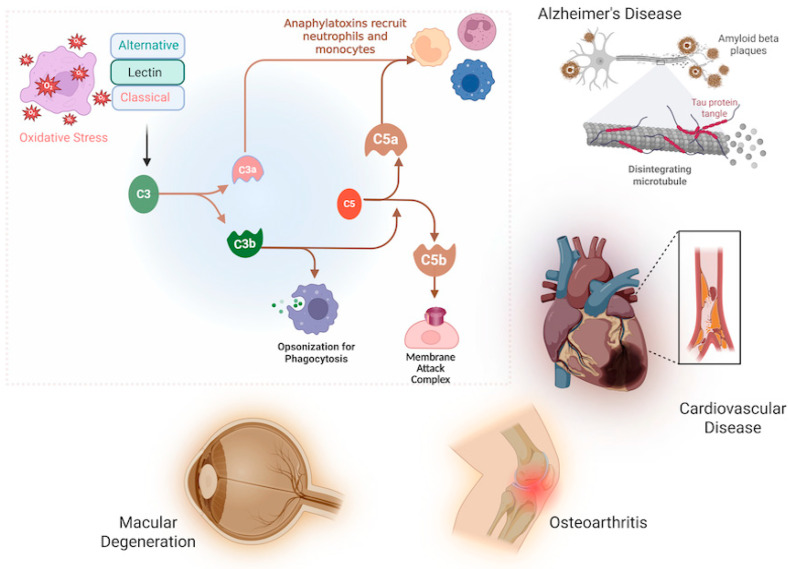
Schematic overview of complement system activation and its role in neurodegenerative and other chronic diseases. The figure illustrates the classical pathway of complement activation and its downstream effects, linking this complement activation to several disease states, including: Alzheimer’s disease, associated with oxidative stress, tau protein tangles, and disintegrating microtubules, cardiovascular disease, macular degeneration, and osteoarthritis.

The complex phenomenon of aging makes it hard to understand, but inflammation remains as the key factor to the oddities associated with aging [200]. It is known that aging and the complement system have close interrelations, however, the relationship remains unclear. The complement cascade affects inflammation, apoptosis, metabolism, and other organelle functions as the complement components are found in the circulation, body fluids, and tissues. The complement system plays a fundamental role in defense mechanisms mediated by various endogenous and exogenous stimuli that may cause injury to cells. However, recently, the view of the complement system has changed and it is considered as a chief system that connects several responses during immune and inflammatory reactions and not merely restricted to elimination of pathogens (Figure 6). Uncontrolled activation of the complement cascade results in undesired damage to tissues. There is a mutual relationship between the complement system and pro-inflammatory cytokines (Figure 6). Studies show that pro-inflammatory cytokines are associated with increased expression of anaphylatoxin receptors in inflammatory cells [201]. Moreover, aging is most commonly associated with diminished immunity. As a result, the ability of elderly populations to battle against foreign pathogens is also diminished and they are thus more prone to infectious diseases [202] and increased incidence of autoimmune disorders [203]. There are several important age-related alterations observed in the complement system. It is important to consider these changes when understanding complement-associated diseases.

### 9.1. Complement and Age-Associated Inflammatory Diseases

In general, when inflammation is involved in any pathological processes, complement has to be taken into account as a possible mediator in the disease progression [204]. The complement system is activated upon ischemia and reperfusion and considered as a major contributor causing tissue damage. Likewise, complement activation occurs during ischemia/reperfusion of the intestine [205], sepsis [206], hemorrhagic shock [207], pulmonary injury [208], and myocardial infarction [209]. Complement activation is also observed in immune-complex diseases like rheumatoid arthritis, systemic lupus erythematosus, systemic inflammatory response syndrome (SIRS), inflammatory and degenerative diseases in the nervous system, inflammatory problems post cardiopulmonary bypass and hemodialysis, arteriosclerosis, acute respiratory distress syndrome (ARDS), trauma, septic shock, and many more [210]. The terminal complement complex also stimulates platelet activation leading to platelet–leukocyte aggregation [211]. C3a and C5a are potent anaphylatoxins that play a major role in activating leukocytes and endothelial cells. C5a, being more potent than C3a, is involved in enhancing the expression of adhesion molecules on the endothelium and inducing the release of several important cytokines like IL-6, TNF-α, and monocyte chemoattractant protein-1 [212,213,214]. Also, C5a is a solid chemoattractant associated with the increased accumulation of inflammatory cells at the primary site of injury [212]. Animal studies support the functional relationship between activation of the complement cascade and the pathological process of thrombosis and atherosclerosis [215]. Generally, the complement is measured as a serum component, but its components are also found in the synovial fluid of joints at high concentrations and upon cartilage injury. The complement components in synovial fluid get exposed to extracellular matrix (ECM) components released from cartilage by proteinases [216]. However, during arthritis, along with many ECM components, several other complement activators with dying cells and autoantibodies are present, resulting in alterations in the balance between complement activation and inhibition, leading finally to the release of C5a anaphylatoxin and formation of the membrane attack complex, thus reconstituting a solid inflammatory milieu [217].

### 9.2. The Complement System and Selected Age-Associated Diseases

The complement system is a vital component of humoral immunity, with roles in both innate and adaptive immunogenicity responses [218]. Its dysregulation is a common feature in the pathogenesis of numerous age-related diseases. Table 1 below summarizes the key relationships between oxidative stress, complement activation, and specific age-related pathologies, highlighting the recurrent theme of complement-mediated inflammation amplifying underlying oxidative damage.

#### 9.2.1. The Complement System and Age-Associated Cardiovascular Diseases

The pro-inflammatory state of aging contributes to insulin resistance and dyslipidemia, driving cardiovascular disease [230]. Complement activation is an established feature of acute myocardial infarction, with components C1q, C3, C4, and C5b-9 deposited in ischemic myocardium [66,68,231]. Beyond acute injury, the anaphylatoxin C5a has been shown to predict future cardiovascular complications in patients with peripheral arterial disease, independent of traditional risk factors, solidifying the role of chronic complement activation in atherosclerotic progression [220,221].

#### 9.2.2. The Complement System and Age-Associated Alzheimer’s Disease

The role of complement in Alzheimer’s disease (AD) is complex, balancing protective clearance and pathological inflammation [232]. Biochemical assays demonstrate that fibrillar Aβ and aggregated tau activate the classical and alternative pathways, driving local deposition of C1q, C3, and the MAC in parenchymal plaques [222,233]. This activation occurs early in disease progression, before significant gliosis and neurodegeneration [233]. Critically, studies using C5a receptor antagonists in AD transgenic mice showed reduced gliosis, less fibrillar amyloid, and improved behavioral outcomes, providing direct evidence for the pathogenic role of complement-mediated inflammation in AD [224].

#### 9.2.3. The Complement System and Age-Associated Osteoarthritis

In osteoarthritis (OA), complement activation is a key mediator of synovial inflammation and cartilage degradation. Metabolic reprogramming of synovial fibroblasts stimulates a potent inflammatory response that is significantly driven by C3 and C3a [225]. Components C3a, C5a, and the MAC are present in the synovial fluid of OA patients, and their levels are elevated compared to controls [226]. The pathological significance is confirmed by experimental models, where knockdown of C3 reduces inflammatory damage and mice deficient in C5 show decreased inflammation in chondrocytes, highlighting the complement system as a central driver of OA pathology [225].

#### 9.2.4. The Complement System and Age-Associated Macular Degeneration

Age-related macular degeneration (AMD) is strongly linked to genetic mutations in complement regulators like factor H and factor I, leading to dysregulation of the alternative pathway and diminished control of C3 activation [227,229]. This results in the accumulation of complement-activating immune deposits (drusen) containing C3a and C5a between the retinal pigment epithelium and Bruch’s membrane [234,235]. The ratio of C3d/C3 is significantly higher in AMD patients, indicating sustained complement activation [228]. This inflammatory milieu promotes choroidal neovascularization in wet AMD and drives the atrophy of retinal cells in geographic atrophy, making the complement pathway a primary therapeutic target [235].

## 10. Therapeutic Relevance

Since the last decade, the focus on the complement system has been increasing, as uncontrolled activation has shown a close association with a variety of clinical disorders like thrombotic, hemolytic, and autoimmune disorders affecting a wide range of organs. Modulating the complement system with anti-complement agents is now considered as a promising and novel etiology for treating several human diseases (Table 2) [236,237,238]. The approval and therapeutic achievement of the first anti-complement agent, eculizumab, which is a monoclonal antibody that interferes with the cleavage of the C5 complement component [239], have shown its potential impact for treating paroxysmal nocturnal hemoglobinuria (PNH) [240,241,242] and atypical hemolytic uremic syndrome (aHUS) [243]. Another C5-specific monoclonal antibody, ravulizumab, provides potential, immediate, and sustained C5 inhibition by preventing the formation of C5a and C5b, thus preventing complement activation and hemolysis, providing better PNH and aHUS treatment outcomes [244]. The wide-ranging effects of C5 inhibitors on the terminal pathway of the complement system, lacking specificity, may expose patients to increased infections. Therefore, development of complement inhibitors that are pathway-specific has been a thought-provoking goal. Compstatin, compstatin 40 (Cp40), and polyethylene glycol (PEG-Cp40), a long-acting analog of Cp40, are some of the recently developed complement inhibitors effective on the upstream processes of the complement cascade [245]. Presently, Cp40 and PEG-Cp40, compstatin linked inhibitors, are used for treating patients with PNH [246]. Other monoclonal-antibody-derived pathway-specific inhibitors include TNT003, sutimlimab, and H17/3E7. Studies suggest that TNT003 is effective against serine proteinase C1s and inhibits complement deposition and formation of terminal complement complexes, preventing intravascular and extravascular hemolysis in cold agglutinin disease (CAD) patients [247]. Similarly, sutimlimab also binds to C1s and blocks hemolysis and corrects anemia and removes the need for blood transfusion in CAD patients [248]. H17/3E7 inhibits erythrocyte lysis in PNH patients and is effective against formation of C3 and C5 convertase by combining with C3 and C3b complement components, inhibiting activation of the complement alternative pathway [249]. Other pathway-specific inhibitors are recombinant fusion proteins, which include TT30 and CRIg-FH/CRIg-L-FH. Studies have shown that TT30 inhibits C3b-mediated extravascular hemolysis in PNH and the alternative-pathway-mediated C3 deposition on erythrocyte membranes, inhibiting intravascular and extravascular hemolysis of autoimmune hemolytic anemia (AIHA) [250]. CRIg-FH and CRIg-L-FH are CRIg-targeted complement inhibitors that prevent classical- and alternative-pathway-mediated hemolysis by eliminating the deposition of C3b/iC3b in PNH patients [251].

Enrichment in antioxidant defenses through nutritional supplements seems to provide an additional realistic and practical approach to minimize the level of oxidative stress [261]. However, few successful reports have been made about using antioxidant therapy in mammalian models of diseases induced by oxidative stress [262]. Moreover, randomized clinical trials in humans have failed to demonstrate any clinical benefit from antioxidant supplementation [263]. Therefore, successful implementation of this strategy may require deeper understanding of the pharmacological aspects like absorption rate, distribution, and metabolism of different agents being used. Development of novel compounds that mimic SOD and catalase has shown a promising alternative approach [144,264,265,266]. Such compounds have been found to be effective in decreasing oxidative-stress-related diseases [265,266].

## 11. Conclusions

Aging is characterized by the progressive accumulation of oxidative damage and a state of chronic, low-grade inflammation. As this review illustrates, these two hallmarks are not independent but are powerfully linked through the activation of the complement system. Evidence from both clinical observation and experimental models demonstrates that oxidative stress serves as a key trigger for complement activation via multiple pathways, while complement-derived anaphylatoxins and the MAC subsequently fuel further ROS production and inflammatory cell recruitment. This creates a self-perpetuating cycle that accelerates cellular senescence, tissue dysfunction, and the progression of age-related diseases, from cardiovascular and neurodegenerative disorders to macular degeneration.

Our work, identifying the Rpl13a snoRNA/NADPH oxidase/ROS pathway in red blood cells as a mechanism driving complement C3 deposition and thrombosis in aging, provides a specific example of this vicious cycle at play in the vasculature [27]. This mechanistic insight, alongside the broader evidence presented, underscores the therapeutic potential of disrupting this cycle.

The development of novel complement inhibitors, such as C5 monoclonal antibodies (eculizumab, ravulizumab) and upstream inhibitors like compstatin, offers a promising avenue for treating complement-driven age-related pathologies [236,237,238]. Future research should focus on defining the precise oxidative stress triggers in different tissues and evaluating the efficacy of targeted anti-complement and antioxidant strategies in ameliorating the specific pathologies of aging. However, while targeting the oxidative-stress–complement axis may prove instrumental in not only treating age-related diseases but also in promoting healthier aging, the major limitation is the lack of mechanistic studies directly linking ROS-driven complement activation to specific age-related diseases.

Most current evidence is correlative and does not establish clear cause-and-effect pathways. For instance, while elevated levels of complement proteins and oxidative markers are often found concurrently in aged or diseased tissues, it is unclear whether oxidative stress is the primary instigator of complement activation or if complement activation itself generates a pro-oxidant environment, creating a vicious cycle that is difficult to disentangle in human observational studies. Additionally, the interplay between ROS, complement regulators, and immune–metabolic pathways in aged tissues remains poorly defined. The impact of age-related epigenetic changes and cellular senescence on the expression and function of key regulators like factor H is a critical unknown, leaving a gap in understanding how homeostatic control is lost. Furthermore, the limited availability of age-appropriate in vivo models—such as animal models that accurately recapitulate the slow, multisystemic nature of human aging—further restricts accurate interpretation of these complex interactions. Most experiments rely on acute injury or genetically modified young animals, which may not reflect the chronic, low-grade inflammation characteristic of human aging. Overall, these gaps make it challenging to fully understand the temporal sequence and relative contribution of ROS-mediated complement activation to aging and disease progression, thereby hindering the development of targeted therapeutic interventions that can safely modulate this axis without compromising essential immune functions.

## Figures and Tables

**Table 1:** Interplay of Oxidative Stress and Complement Activation in Age-Related Pathologies.

Disease	Key Oxidative Stress Link	Complement System Involvement	Key Evidence
Cardiovascular Disease	Endothelial dysfunction, atherosclerosis	C3/C5a predict events; MAC deposition	C5a levels predict complications; C3 elevated in patients [219,220,221]
Alzheimer’s Disease	Amyloid-beta and tau pathology	C1q, C3, C5b-9 found in plaques; C5aR activation	C5aR antagonists reduce pathology in mouse models [222,223,224]
Osteoarthritis	Cartilage degradation, chondrocyte stress	C3a, C5a, MAC in synovial fluid; C3 drives inflammation	C3 knockdown reduces damage; components found in joint [225,226]
Macular Degeneration	Retinal pigment epithelium damage	Alternative pathway dysregulation; C3/C5a in drusen	Genetic mutations in C3, FI; elevated C3d/C3 ratio [227,228,229]

**Table 2:** Complement-Targeted Therapeutics.

Drug Name	Target	Mode of Action	Key Indications	Refs
Eculizumab	C5	mAb; inhibits C5 cleavage → prevents MAC formation	PNH, aHUS	[252]
Ravulizumab	C5	Long-acting mAb; inhibits C5 cleavage → prevents MAC formation	PNH, aHUS	[253]
Pegcetacoplan	C3	Binds C3; inhibits C3 activation and downstream cascade	PNH, geographic atrophy (AMD)	[254,255]
Iptacopan	Factor B	Inhibits factor B; blocks alternative pathway activation	PNH, IgA nephropathy	[256,257]
Sutimlimab	C1s	mAb; inhibits C1s → blocks classical pathway activation	Cold agglutinin disease (CAD)	[257,258]
Avacopan	C5aR1	C5a receptor antagonist; inhibits neutrophil inflammation	ANCA-associated vasculitis	[259,260]

## Data Availability

No new data were created or analyzed in this study. Data sharing is not applicable to this article.
